# Increased Serum C-reactive Protein and Corpus Callosum Alterations in Older Adults

**DOI:** 10.14336/AD.2018.0329

**Published:** 2019-04-01

**Authors:** Fabienne Cyprien, Philippe Courtet, Jerome Maller, Chantal Meslin, Karen Ritchie, Marie-Laure Ancelin, Sylvaine Artero

**Affiliations:** ^1^INSERM, Univ Montpellier, Neuropsychiatry, Epidemiological and Clinical Research, Montpellier, France.; ^2^CHU Montpellier, F-34095, France.; ^3^Monash Alfred Psychiatry Research Centre, Central Clinical School, Monash University and Alfred Hospital, Melbourne, Australia.; ^4^Centre for Research on Ageing, Health and Wellbeing, Research School of Population Health, ANU College of Medicine, Biology and Environment at the Australian National University, Canberra, Australia.

**Keywords:** corpus callosum, C-reactive protein, magnetic resonance imaging, older people, inflammation

## Abstract

Chronic systemic low-grade inflammation is associated with aging, but little is known on whether age-related inflammation affects brain structure, particularly white matter. The current study tested the hypothesis that in older adults without dementia, higher serum levels of high-sensitivity C-reactive protein (hs-CRP) are associated with reduced corpus callosum (CC) areas. French community-dwelling subjects (ESPRIT study) aged 65 and older (N=101) underwent hs-CRP testing and structural magnetic resonance imaging (MRI). Multiple linear regression models were carried out. In the unadjusted model, higher hs-CRP level was significantly associated with smaller anterior, mid, and total midsagittal CC areas, but not with the posterior CC area. These associations were independent of demographic characteristics and intracranial volume. After adjustment for body mass index, diabetes, inflammation-related chronic pathologies and white matter lesions (WML), only the associations between hs-CRP level and smaller anterior and total midsagittal CC areas were still significant, although weaker. These findings suggest that low-grade inflammation is associated with CC structural integrity alterations in older adults independently of physical or neuropsychiatric pathologies.

In humans, aging is associated with chronic systemic low-grade inflammation that is increasingly defined as "inflammaging" [1]. High-sensitivity C-reactive protein (hs-CRP) is a sensitive marker of systemic low-grade inflammation [2] that can be routinely measured. This acute-phase protein is synthesized by liver in response to inflammation, and has been identified as an independent predictor of stroke and heart attack [3], and of dementia diagnosis[4]. It has also been linked to frailty [5], functional and global cognitive decline [6], and psychiatric disorders [7].

Magnetic resonance imaging (MRI) could help bringing insights into the link between inflammatory markers and cognitive function. The few existing studies on the relation between hs-CRP level and brain structure in older adults have consistently reported the association of higher CRP level with reduced hippocampal and gray matter volume [8], cortical thinning [9] and total brain atrophy [10]. Moreover, analysis of the brain white matter (WM) microstructure by diffusion tensor imaging (DTI) showed that higher CRP levels are associated with WM microstructural disintegration, particularly in the frontal and temporal lobes [11, 12] and corpus callosum (CC) [12, 13].

CC is the main commissure between the cerebral hemispheres. It contains between 200 and 800 million axon fibers and is of crucial importance for interconnecting associative brain areas that play a pivotal role in the integration of inter-hemispheric information and higher cognitive functions. CC alterations may lead to cognitive and emotional deficits [14], and some links with neuropsychiatric disorders have been demonstrated. Particularly, CC abnormalities have been reported in neurodegenerative diseases [15], mood disorders [16, 17] and suicidal behavior [18]. Moreover, the CC is one of the first regions to show signs of aging-related degeneration [19]. Recently, we showed that smaller CC size is predictive of incident late-life depression over 10 years in older community-dwelling women, independently of cognitive deterioration [17].

Despite the association between CC alterations and mental illness occurrence in older adults, the underlying mechanisms have not been clarified yet. Specifically, the association between hs-CRP level and CC microstructure integrity in older adults without dementia has not been investigated. Indeed, only one study specifically assessed hs-CRP level in relation to DTI imaging parameters [12], including the CC, but none have focused on the investigation of hs-CRP and CC by using structural MRI data.

Therefore, the objective of this study was to test whether in a community sample of older adults (from 65 to 80 years of age) without dementia, higher hs-CRP serum levels are associated with reduced size of specific CC sub-regions calculated from brain structural MRI images, by taking into account also potential confounding factors, such as physical and neuropsychiatric comorbidities.

## MATERIAL AND METHODS

### Participants

The data used for this analysis were from a longitudinal study on neuropsychiatric disorders in older adults in France (ESPRIT study) [20]. For the ESPRIT study, participants (aged 65 and over) were randomly selected from the electoral rolls of Montpellier, France, between 1999 and 2001. At inclusion, they were interviewed at the study center or in their own home, if disabled. Standardized interviews, neuropsychological tests and neurological examination were carried out at baseline. The study protocol was approved by the Bicêtre University Hospital Ethical Committee (France) and written informed consent was obtained from each participant.

For the present study, individuals from the ESPRIT cohort (n=1863) were pre-selected using the following criteria: age ≤80 years, right-handedness, availability of MRI data with estimation of the size of CC sub-regions and total brain volume (n=710). From this initial group, individuals who received a diagnosis of dementia (n=17) were excluded. Among the remaining participants, only 104 underwent hs-CRP testing. Thus, the present analysis concerned these 104 participants.

#### MRI imaging analysis

##### Corpus callosum measurement

A Signa 1.5T GE Imaging System (General Electric Medical Systems, Milwaukee, Wisconsin) was used to acquire contiguous anterior commissure-posterior commissure, aligned, axial inversion recovery-prepared, spoiled gradient recalled, T1-weighted sequences for volumetric estimations (TR = 12, TE = 2.8, inversion time = 600, matrix size = 256×256, pixel spacing = 0.9375×0.9375 mm^2^, number of excitations = 1, slice thickness = 1.0 mm). Slices were then converted to isotropic images (0.9375 mm^3^) and re-sliced to 1.00 mm^3^. The CC outline was manually traced on the midline sagittal slice of the T1-weighted images, using anatomical landmarks in a hierarchical order [21] and the region-of-interest module of Analyze 9.0 (Brain Imaging Resource, Mayo Clinic, Rochester, Minnesota) on a Windows XP Professional workstation (Microsoft, Redmond, Washington). The landmarks based on the midline sagittal slice were: (1) no WM or only minimal WM in the cortical mantle surrounding the CC; (2) the interthalamic adhesion; and (3) the transparent septum and the visibility of the aqueduct of Sylvius. Using landmarks adapted from Witelson’s method, the manually segmented CC outline was automatically divided in three sub-regions, as previously described [22]. This approach originated from autopsy studies and was based on the topographic distribution of CC fibers. It is probably robust relative to age-related changes and takes the CC global curvature into account [23].

Specifically, the traced CC outline was vertically divided in three equal-length parallel horizontal divisions (sub-regions; CC1 to CC3; CC1 incorporates the rostrum and genus, and CC3 is the splenium) using the division feature (and Grid option) within the region-of-interest module of the Analyze software, and then the area (expressed in mm^2^) within each division was calculated.

##### Estimation of the white matter lesion (WML) volume

To take into account the possible WML confounding effect on CC size [23], WML volume (to be used as a covariate) was also estimated using a semi-automatic method described in detail elsewhere [24]. WML values were transformed using a log_10_ (*x*+0.01) function because of their highly asymmetric distribution and possible null values.

##### Intracranial volume (ICV) estimation

The intracranial volume (gray plus white matter plus cerebrospinal fluid) was used as a covariate in the models to minimize the effect due to global brain size differences. It was computed using the segment m-file of the SPM5 software (Wellcome Department of Cognitive Neurology, London, United Kingdom).

#### Socio-demographic and clinical parameters

The standardized interview included items on demographic characteristics, education level (no formal education or primary school, lower secondary education, higher secondary education, and university degree), height, weight, smoking, and alcohol consumption. Cognitive function was assessed with the Mini Mental State Examination (MMSE) questionnaire [25], and the current level of depressive symptomatology was evaluated with the Center for Epidemiologic Studies Depression Scale (CESD), validated in the older people [26]. The body mass index (BMI) was calculated as follows: [weight (kg)/height (m)^2^] and analyzed as a continuous variable.

The history of cardiovascular disease (stroke, myocardial infarction, angina pectoris, arteritis or coronary surgery) was established using standardized questionnaires and additional information, if needed, from general practitioners. Self-reported respiratory disorder (chronic bronchitis, asthma or dyspnea) and regular treatment for chronic joint or back pain were considered as indicative of inflammation-related chronic pathologies. All drugs used in the previous month, including anti-inflammatory medications were recorded from medical prescriptions, drug packages and any other relevant information.

Blood pressure was measured twice during the interview using an OMRON M4 digital electronic tensiometer. Subjects were considered as having hypertension when the mean of the two measurements was ≥160/95 mm Hg, or if they were taking anti-hypertensive drugs. Fasting blood samples were taken to determine the cholesterol and glucose levels. Hypercholesterolemia was defined as cholesterol sulfotransferase (CST) ≥6.2 mmol/l, and diabetes as fasting blood glucose ≥7mmol/l or taking diabetes treatment.

**Table 1:** Characteristics of the study population.

**Demographical/Clinical characteristics**	
Participants, n	101
Women, %	54.5
Age, years (mean ± SD [min-max])	71.4 ± 4.1 (65-80)
Education level, % (n)	
No formal education or primary school	22.8 (23)
Lower secondary education	28.7 (29)
Higher secondary education	24.8 (25)
University degree	23.8 (24)
MMSE^a^, median (IQR)	27 (3)
Depressive symptomatology^b^, % (n)	22.8 (23)
Current or former smoker, % (n)	46.5 (46)
Alcohol consumption^c^, % (n)	40.6 (41)
Body Mass Index^d^, kg/m^2^ (mean ± SD [range])	24.4 ± 3.3 (18.0-32.2)
History of cardiovascular disease^e^, % (n)	5.0 (5)
Hypertension^f^, % (n)	45.5 (46)
Diabetes^g^, % (n)	4.0 (4)
Hypercholesterolemia^h^, % (n)	50.5 (51)
Inflammation-related chronic pathologies^i^, % (n)	29.7 (30)
C-reactive protein, mg/L (mean ± SD [range])	2.6 ± 0.2 (0.2-9.2)
**Structural imaging**	
Corpus callosum areas, mm^2^ (mean ± SD)	
Anterior	220.4 ± 4.0
Mid	136.2 ± 3.0
Posterior	237.6 ± 3.9
Total area	594.1 ± 9.5
Intracranial volume, cm^3^ (mean ± SD)	1207.6 ± 14.5
White matter lesions volume, mm^3^ [median (IQR)]	0.90 (2.8)

a Mini Mental State Examination

b Center for Epidemiologic Studies Depression Scale, score ≥16

c Alcohol consumption ≥12g/day

d Weight (kg)/height (m^2^)

e History of stroke, myocardial infarction, angina pectoris, arteritis or coronary surgery

f Systolic blood pressure ≥160 mm Hg and/or diastolic blood pressure ≥95 mm Hg or use of antihypertensive medication

g Treatment for diabetes or glucose ≥7 mmol/L

h Total cholesterol ≥6.2 mmol/L

i Self-reported respiratory disorder (chronic bronchitis, asthma or dyspnea) or taking regular treatment for chronic joint or back pain

#### Circulating CRP

For hs-CRP testing, thawed serum separated from blood samples collected after 12h fast and a particle enhanced immunoephelometry assay were used. The sensitivity and inter-assay coefficient variation were 0.17 mgl^-1^ and 2.3%, respectively. People with hs-CRP levels >10 mg/l were excluded from the analyses, because this high level was considered to be due to acute-phase response (n=3). A logarithmic transformation was used for statistical analysis due to the highly asymmetric distribution of hs-CRP values.

#### Statistical analysis

The Pearson correlation coefficient and Student’s *t* test were used to assess associations between hs-CRP values and clinical and imaging variables. To assess the relationship between hs-CRP level and CC areas, three sets of multiple linear regression models were performed. First, unadjusted regression (correlation) coefficients were calculated (model 1). The second model was adjusted for age, sex, ICV, and education level. The third model took into account all possible confounders and mediators by adjusting for factors that were significantly associated with hs-CRP level in the crude analyses (*P*<.05). Statistical analyses were carried out with IBM SPSS Statistics (SPSS Inc., Chicago, Illinois, USA, version 20).

## RESULTS

The demographic and clinical characteristics of the study population are summarized in Table 1. The participants’ mean age was 71 years, 54.5% were women, and the median hs-CRP serum level was 2.6 mg/l.

### Associations between hs-CRP level and covariates

The log-transformed hs-CRP value increased significantly with BMI (*r*=.381, *P*<.0001) and the log-transformed WML (*r*=.299, *P*=.002). Higher levels of serum hs-CRP were significantly associated with history of diabetes (*P*=.016) and history of inflammation-related chronic pathology (P=.001). There was no significant association with age (*r*=.062, *P*=.539), sex (*P*=.964), education level (*P*=.462), MMSE score (*r*=-.122, *P*=.223), smoking (*P*=.911), alcohol consumption (*P*=.845), history of cardiovascular disease (*P*=.203), hypertension (*P*=.689), hypercholesterolemia (*P*=.833), and depressive symptomatology (*P*=.725).

**Table 2:** Association of serum hs-CRP level with corpus callosum area.

Corpus callosum region	model 1			model 2			model 3		
	β	Standard error	p	β	Standard error	p	β	Standard error	p
Anterior area	-0.302	0.001	0.002	-0.372	0.001	0.001	-.303	0.001	0.003
Mid area	-0.257	0.001	0.010	-0.272	0.001	0.010	-.182	0.001	0.054
Posterior area	-0.137	0.001	0.171	-0.153	0.001	0.151	-.098	0.001	0.291
Mid-sagittal total area	-0.268	<0.0001	0.007	-0.311	<0.0001	0.004	-.228	<0.0001	0.020

Model 1: unadjusted

Model 2: model adjusted for age, sex, intra-cranial volume, and educational level

Model 3: model 2 + BMI, log WML, diabetes, inflammation-related chronic pathologies as covariates

### Associations between hs-CRP level and corpus callosum areas measured by MRI

**Figure 1.**
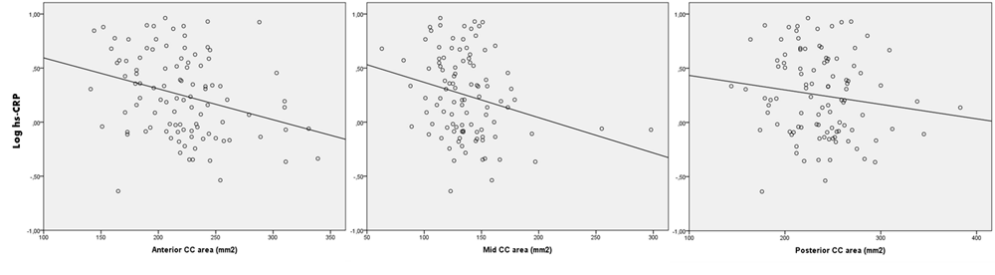
Slopes showing the association between log hs-CRP serum level and the anterior, mid, posterior corpus callosum (CC) areas (unadjusted).

The regression analyses (Table 2) showed that higher hs-CRP level was significantly associated with smaller anterior, mid and total midsagittal CC areas, but not with the posterior CC area (Fig. 1). These associations were still significant after adjustment for demographic characteristics and ICV (model 2). After further adjustment for BMI, diabetes, inflammation-related chronic pathologies and log WML, only the association with smaller anterior and total midsagittal CC areas remained significant (model 3). In supplementary analyses (model 4, not shown), addition of depression (CESD score) to model 3, although non-significant in the crude analysis, did not change the results. In other supplementary analysis, we have eliminated the 6 subjects who developed incident Alzheimer’s disease at 4-year follow-up in the aim to test the link between CC and CRP independently of dementia. These analyses did not change the results (data not shown).

## DISCUSSION

To our knowledge, this is the first study to examine the relationship between hs-CRP serum levels and CC areas in a community sample of non-demented subjects aged 65 to 80 (n=101). We found that higher hs-CRP levels were associated with smaller anterior, mid and total midsagittal CC areas. These associations were not weakened after adjustment for age, sex, education level, ICV and depression. However, after controlling for cardiovascular risk factors and WML, the association was weakened for the anterior and total midsagittal CC areas and lost for the mid CC area.

The negative association between hs-CRP serum level and total mid-sagittal CC area in community-dwelling older people suggests that this main interhemispheric commissure may be vulnerable to late-life inflammation-associated alterations. Moreover, cardiovascular risk factors and WML seem to partly account for the association, differently from age, sex, education level and ICV. By analyzing each CC sub-region, we found that the strongest and most significant association was between hs-CRP level and the anterior CC, even after controlling for potential moderating factors.

Literature data on CC and CRP in older subjects are scarce and limited to DTI measurements for calculation of WM integrity quantified by fractional anisotropy (FA), thus making difficult the comparison with our findings. High hs-CRP levels have been related to lower FA values for CC, particularly the genu [12]. This is consistent with a study showing that a composite measure of circulating inflammatory markers (CRP and tumor necrosis factor-alpha) was negatively correlated with the FA values of the CC body and isthmus in a community sample of 95 subjects older than 70 years of age and without dementia [13]. Bettcher et al [27] specifically focused on CC integrity and age but used interleukin-6 (IL-6), and not CRP, as a marker of inflammation. They found an inverse association between IL-6 levels and CC FA values. Although the comparison between studies is limited given the huge methodological differences, our results are consistent with the few data on the association between CC integrity and inflammatory markers.

Our findings also suggest that vascular factors could partly influence the association between hs-CRP level and CC size during aging, but for the anterior CC area that remained correlated with hs-CRP level after adjustment. However, given the overlapping and often controversial role of vascular and inflammatory markers, their relative contribution is difficult to distinguish especially because hs-CRP can be interpreted both as an inflammatory and vascular disease blood marker [28, 29]. Moreover, we did not investigate whether vascular risk factors influenced the relationship between hs-CRP level and WM integrity in CC. Previous studies have linked CC alterations to different vascular/inflammatory patterns [12, 30] that may reflect regional CC susceptibility. For instance, degenerative processes have a more prominent influence than vascular processes on splenium structural integrity [30]. This is in accordance with our finding that the association between hs-CRP level and anterior and mid CC areas is modulated by cardiovascular risk factors and WML. Conversely, hs-CRP level was not associated with the splenium (posterior) area even in the unadjusted model, possibly because people with neurodegenerative diseases were not included in the analyses. Conversely, other studies have hypothesized that inflammation is increased by oligodendrocyte damage, resulting from pathological aging, and by the loss of the trophic support provided by these cells [31]. Importantly, our findings suggest that the analysis of the association between hs-CRP level and CC integrity without considering WML, cardiovascular factors, and inflammation-related chronic pathologies could yield an inaccurate model.

There are some limitations inherent to the study design. The sample was small (n=101) because not all people included in the ESPRIT cohort (n=1863) had CRP and MRI measurements. This may have underpowered the ability to perform moderation and mediation analyses that could have helped to more precisely elucidate the effect of potential confounders, such as cardiovascular risk factors and WML, on the relationship between hs-CRP and CC area size. It may also have underpowered the effect of cardiovascular risk factors and WML in our regression models, because the association strength was only reduced for the anterior and total CC areas, whereas the association was lost for the mid CC area. Finally, the study focused on one single non-specific peripheral inflammatory marker the level of which could be affected by underlying infections or diseases. However, the exclusion of all subjects with hs-CRP level >10 mg/l makes unlikely the attribution of the observed results to the acute phase inflammation response.

This study suggests that the association between higher hs-CRP levels and lower CC areas in community-dwelling older adults without dementia is a sign of “inflammaging”, possibly caused by low-grade chronic upregulation of some inflammatory responses associated with aging. This association appears to be partly influenced by cardiovascular risk factors and WML. These findings bring new insights into the mechanisms underlying alterations of the interhemispheric connectivity in normal aging, highlighting the important role of chronic inflammation. Larger studies are needed to precisely estimate the contribution of inflammation to WM microstructure alterations in healthy and pathological aging.
